# Supplementary material for: Knowledge about cervical cancer and awareness about human papillomavirus vaccination among medical students in Jordan
Source: PeerJ. 2021 Jun 17;9:e11611. doi: 10.7717/peerj.11611 (PMC8214844; doi:10.7717/peerj.11611)
Supplement: Supplemental Information 2 [file peerj-09-11611-s002.doc]

**Awareness about cervical cancer among Medical University students in Jordan**

____________________________________________________________

Dear Students

Researchers from the Faculty of Pharmacy and Faculty of medicine/ Yarmouk and JUST University are carrying out a project to assess knowledge and awareness about cervical cancer and HPV vaccination among Medical University students in Jordan. This Survey is developed for research purpose and not for the critical appraisal of students.

Your participation in completing this survey is highly appreciated.

□ Agree

□ Disagree

If you agree to patriciate please answer the questions without referral to consulting materials, textbooks or fellow staff __________________________________________________________________________

Part 1: Student information

| **Student gender**:   - Male - Female | **University:** |
| --- | --- |
| **Age**:_______ years | **Level:**   - 3^rd^ year - 4^th^ year - 5^h^ year - 6^th^ year |
| **Family income:** | **Place of living:** |
| **Do you Know someone with cervical cancer:**   - Yes - No | **Nationality:** |

**Part 2: Knowledge about cervical cancer and HPV**

1. Cervical cancer is the leading cause amongst gynecological cancer

- Yes
- No
- Do not know

2. The cause of cervical cancer is (Choose one answer)

- Genetic
- Infection
- Environmental

3. The cause of cervical cancer is (Choose one answer)

- Virus
- Bacteria
- Parasite
- Fungus

4. Risk factors of cervical cancer is/are: (CHOOSE as many as applies)

- Multiple sexual partners
- Infection with HPV
- Poor hygiene
- Early age of first coitus
- Smoking
- Family history of the disease
- Multiparty (multiple sex partners)
- Old age
- Contraception (Intrauterine device/oral contraceptives)
- Nulliparity (Never married)

5. Clinical features of cervical cancer include/s: (CHOOSE as many as applies)

| □ No symptoms |  |
| --- | --- |
| □ lower abdominal pain  □ Bleeding per vagina  □ Discharge per vagina |  |
| □ Fever |  |
| □ Menstrual problem |  |
| □ Itching  □ Weight loss  □ Swelling of cervix |  |
| □ Post coital bleeding (spotting or bleeding unrelated to menstruation that occurs during or after sexual intercourse)  □ Anemia | |

6. According to WHO, women aged 25-44 years should be screened every: (Choose one answer)

| □ 2 years  □ 3 years  □ 4 years |  |
| --- | --- |
| □ 5 years |  |

7. According to WHO, women aged 45-60 years should be screened every: (Choose one answer)

| □ 2 years  □ 3 years  □ 4 years |  |
| --- | --- |
| □ 5 years |  |

8. Is there a vaccine protect from cervical cancer?

| - Yes - No - Do not know   9. Does the vaccine protect against all cervical cancer?   - Yes - No - Do not know |
| --- |

10. Girls who have been vaccinated will need to attend for cervical cancer screening

- Yes
- No
- Do not know

11. Is HPV responsible for a wide range of diseases including cervical cancer?

- Yes
- No
- Do not know

12. HPV is transmitted by: (Choose one answer)

- Oro-fecal
- Sexual
- Blood
- Environmental

13. The Technique available for HPV detection: (CHOOSE as many as applies)

- Blood test
- Pap smear
- PCR (Polymerase chain reaction)
- Biopsy

14. HPV subtypes 6 and 11 are commonly associated with: (Choose one answer)

- Genital warts
- Plantar warts
- Cervical carcinoma

| 15. HPV subtypes 16 and 18 are commonly associated with: (Choose one answer)   - Genital warts - Plantar warts - Cervical carcinoma |
| --- |

**Part 3: Awareness and acceptance of HPV vaccination**

1. Is the cervical cancer vaccine available in Jordan?

- Yes
- No
- Do not know

1. Which age group HPV vaccine should be given?

| - 0-10 y | - 10-30y | - 30-50 y | - 50 & above |
| --- | --- | --- | --- |

1. Can it be given to boys?

- Yes
- No
- Do not know

1. Do girls/women need to be screened for HPV before getting vaccinated?

- Yes
- No
- Do not know

1. Can it be given to a woman already having HPV infection?

- Yes
- No
- Do not know

1. How many doses of HPV vaccine are required for protection? (Choose one answer)

| - One | - Two | - Three | - Four |
| --- | --- | --- | --- |

1. Cervical cancer protection provided by HPV vaccine is: (Choose one answer)

| - 100% | - 90% | - 70% | - 50% |
| --- | --- | --- | --- |

1. Is it necessary to introduce HPV vaccine in schoolgirls in Jordan?

- Yes
- No
- don’t know

1. What do you think will be the most important obstacle preventing yourself to receive/advice HPV vaccination? (CHOOSE as many as applies)

| - High cost | - Worry about | - Worry about efficacy of vaccine |
| --- | --- | --- |
| - complications | - Inadequate information |  |

1. What are your sources of knowledge and information on HPV vaccination? (CHOOSE as many as applies)

| - Medical school teachings - Friends | - Newspapers - Books | - Internet - Television |
| --- | --- | --- |

***Thank you for your participation***

***All information obtained in the study will be kept Confidential and used for medical research only***
